# A Multiscale Mathematical Model of *Plasmodium Vivax* Transmission

**DOI:** 10.1007/s11538-022-01036-0

**Published:** 2022-07-01

**Authors:** Md Nurul Anwar, Roslyn I. Hickson, Somya Mehra, James M. McCaw, Jennifer A. Flegg

**Affiliations:** 1 School of Mathematics and Statistics, The University of Melbourne, Parkville, Australia; 2 Department of Mathematics, Bangabandhu Sheikh Mujibur Rahman Science and Technology University, Gopalganj, 8100 Bangladesh; 3 Australian Institute of Tropical Health and Medicine, and College of Public Health, Medical & Veterinary Sciences, James Cook University, Townsville, Australia; 4 Health and Biosecurity, CSIRO, Townsville, Australia; 5 Centre for Epidemiology and Biostatistics, Melbourne School of Population and Global Health, The University of Melbourne, Parkville, Australia; 6 Peter Doherty Institute for Infection and Immunity, The Royal Melbourne Hospital and The University of Melbourne, Parkville, Australia

**Keywords:** Vivax transmission model, Hypnozoite dynamics, Multiscale model, Hypnozoite activation

## Abstract

Malaria is caused by *Plasmodium* parasites which are transmitted to humans by the bite of an infected *Anopheles* mosquito. *Plasmodium vivax* is distinct from other malaria species in its ability to remain dormant in the liver (as hypnozoites) and activate later to cause further infections (referred to as relapses). Mathematical models to describe the transmission dynamics of *P. vivax* have been developed, but most of them fail to capture realistic dynamics of hypnozoites. Models that do capture the complexity tend to involve many governing equations, making them difficult to extend to incorporate other important factors for *P. vivax*, such as treatment status, age and pregnancy. In this paper, we have developed a multiscale model (a system of integro-differential equations) that involves a minimal set of equations at the population scale, with an embedded within-host model that can capture the dynamics of the hypnozoite reservoir. In this way, we can gain key insights into dynamics of *P. vivax* transmission with a minimum number of equations at the population scale, making this framework readily scalable to incorporate more complexity. We performed a sensitivity analysis of our multiscale model over key parameters and found that prevalence of *P. vivax* blood-stage infection increases with both bite rate and number of mosquitoes but decreases with hypnozoite death rate. Since our mathematical model captures the complex dynamics of *P. vivax* and the hypnozoite reservoir, it has the potential to become a key tool to inform elimination strategies for *P. vivax*.

## Introduction

**Fig. 1:**
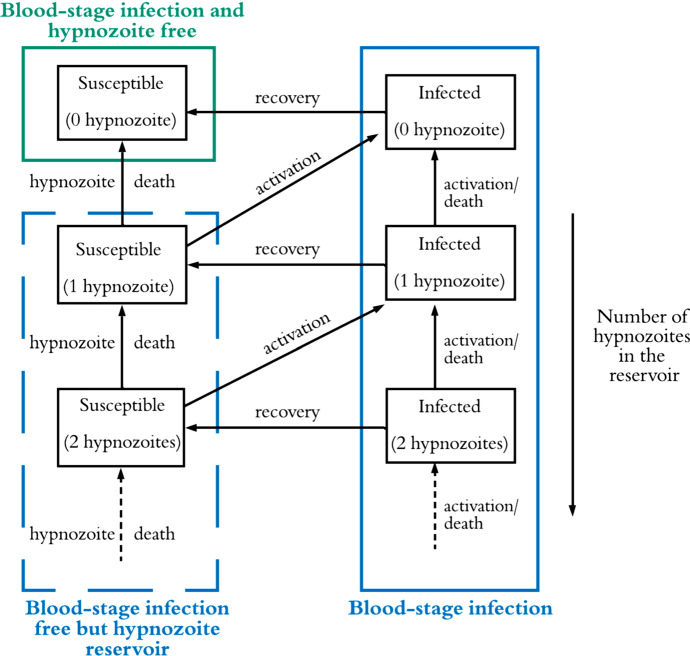
Overview of *P. vivax* disease states and complexity of the hypnozoite reservoir (adapted from White et al. (2014)). Both susceptible and infected individuals may carry hypnozoites within their liver. Activation of a hypnozoite causes a blood-stage infection, while recovery will end the blood-stage infection. The size of the hypnozoite reservoir reduces both with activation and death of a hypnozoite. Not shown explicitly in this schematic is that any individual can be bitten by an infectious mosquito, causing a blood-stage infection and possibly an increase in the size of the hypnozoite reservoir (by one or more hypnozoites). Note that blood-stage infected individuals may or may not carry hypnozoites

Malaria is an infectious disease that poses a significant health threat to humans. Of the malaria parasites, *Plasmodium vivax* is the most geographically widespread and can cause severe infections, resulting in significant associated global morbidity and mortality (Antinori et al. 2012; Battle 2019). In the past, *P. vivax* has been overlooked and mistakenly considered as “benign” (Price et al. 2007), but recent studies have produced evidence that it can cause severe disease (Breman et al. 2007; Kochar et al. 2009; Naing et al. 2014; Tjitra et al. 2008). Of an estimated 241 million malaria cases reported in 2020, *P. vivax* is responsible for 4.5 million cases(World Health Organization 2021). *P. vivax* parasites are transmitted to humans following a bite from an infected mosquito, leading to a (primary) blood-stage infection (Fig. 1). One important characteristic of *P. vivax* transmission is that parasites can remain dormant in the liver for weeks or months (Imwong et al. 2007); these parasites are known as hypnozoites and cause further blood-stage infection, or relapse, upon activation (Fig. 1). It is still not clearly understood what causes hypnozoites to activate (Hulden and Hulden 2011). Hypnozoites might die before activation as a result of the death of the host liver cell (Malato et al. 2011). Both death and activation of hypnozoites reduce the size of the hypnozoite reservoir, see Fig. 1.

The hypnozoite reservoir poses significant complications for *P. vivax* control and elimination (Ferreira and de Oliveira 2015). *P. vivax* is treatable; chloroquine or artemisinin combination therapy (ACT) is currently recommended to treat a blood-stage infection and an anti-hypnozoital drug (e.g. primaquine and tafenoquine) is administered to kill hypnozoites (Asih et al. 2018; Chu and White 2021; Yeung 2012). However, a barrier to the widespread use of anti-hypnozoital drugs is that they cannot be prescribed to all individuals. In particular, primaquine and tafenoquine are not recommended for pregnant and/or lactating people, young children, and those with the genetic condition glucose 6 phosphate dehydrogenase deficiency (G6PDd) that disrupts red blood cell function (Howes et al. 2012; Watson et al. 2018).

The first mathematical model accounting for the effect of hypnozoite relapse on *P. vivax* transmission was introduced by de Zoysa et al. (1988) in a Ross-Macdonald style framework. Later De Zoysa et al. (1991) explicitly modelled up to two hypnozoite broods within a transmission model. Several other mathematical models were later developed for *P. vivax* transmission (Águas et al. 2012; Chamchod and Beier 2013; Ishikawa et al. 2003; Kammanee et al. 2001; Roy et al. 2013; Silal et al. 2019; White et al. 2018, 2014); most consider the hypnozoite reservoir as a single compartment (Águas et al. 2012; Chamchod and Beier 2013; Ishikawa et al. 2003; Kammanee et al. 2001; Roy et al. 2013; White et al. 2014), rather than explicitly accounting for a variable number of hypnozoites in the reservoir. If the size of the hypnozoite reservoir is modelled explicitly, the number of compartments in the model is substantially increased (either truncated artificially or infinite, for example, see Fig. 1) as in White et al. (2014).

Since most *P. vivax* infections are thought to be due to hypnozoite activation rather than new primary infections (Baird 2008; Betuela 2012; Commons et al. 2019, 2018; Luxemburger et al. 1999), incorporating the size of the hypnozoite reservoir in mathematical models is crucial. White et al. (2014) modelled the within-host dynamics of *P. vivax* hypnozoites, considering variability in the size of hypnozoite inoculum across bites, and used the within-host model to parameterise a separate transmission model that captures the full structure of the hypnozoite reservoir (as shown in Fig. 1). That transmission model consists of a set of $2(L_{\text {max}}+1)$ ordinary differential equations (ODEs), where $L_{\text {max}}$ is the maximum number of hypnozoites considered (typically set at 50, giving 102 ODEs). In other work, White et al. have modelled the within-host hypnozoite dynamics with an agent-based model (White et al. 2018), including heterogeneity in exposure to mosquito bites but assuming that hypnozoites established by the same mosquito bite act as a batch and give rise to relapses at the same constant rate. This model does not account for the variability in hypnozoites across bites (White et al. 2018).

Mehra et al. have recently characterised the long-latency hypnozoite dynamics modelled in White et al. (2014) in analytical form (Mehra et al. 2020) with the relaxation of the collective dormancy (hypnozoites established by each mosquito bite progress through the dormancy states as a batch) assumption in White et al. (2014) as the collective dormancy is biologically questionable. Later work by Mehra and colleagues embedded the activation-clearance model governing a single hypnozoite in an epidemiological framework. This framework accounts for continual mosquito bites, where each bite can simultaneously establish multiple hypnozoites (Mehra et al. 2021), and the effect of antimalarial treatment (under a mass drug administration regime) (Mehra et al. 2022). The analytical results from the within-host level can be readily embedded in a population-level model. Embedding a within-host model for hypnozoite dynamics within a simple population-level model allows us to capture, in a single mathematical framework, the complicated *P. vivax* dynamics associated with the hypnozoite reservoir.

In this paper, we embed the within-host model of Mehra et al. (2022) in a simple population-level model for *P. vivax*. By keeping the population-level model simple while capturing the complicated hypnozoite within-host dynamics, an extension of the model to include other important factors will be feasible. The paper is structured as follows. Section 2 describes the development of the multiscale model. In Sect. 3, we provide numerical results of the multiscale model before presenting our discussion in Sect. 4.

## Multiscale Model Development

**Fig. 2:**
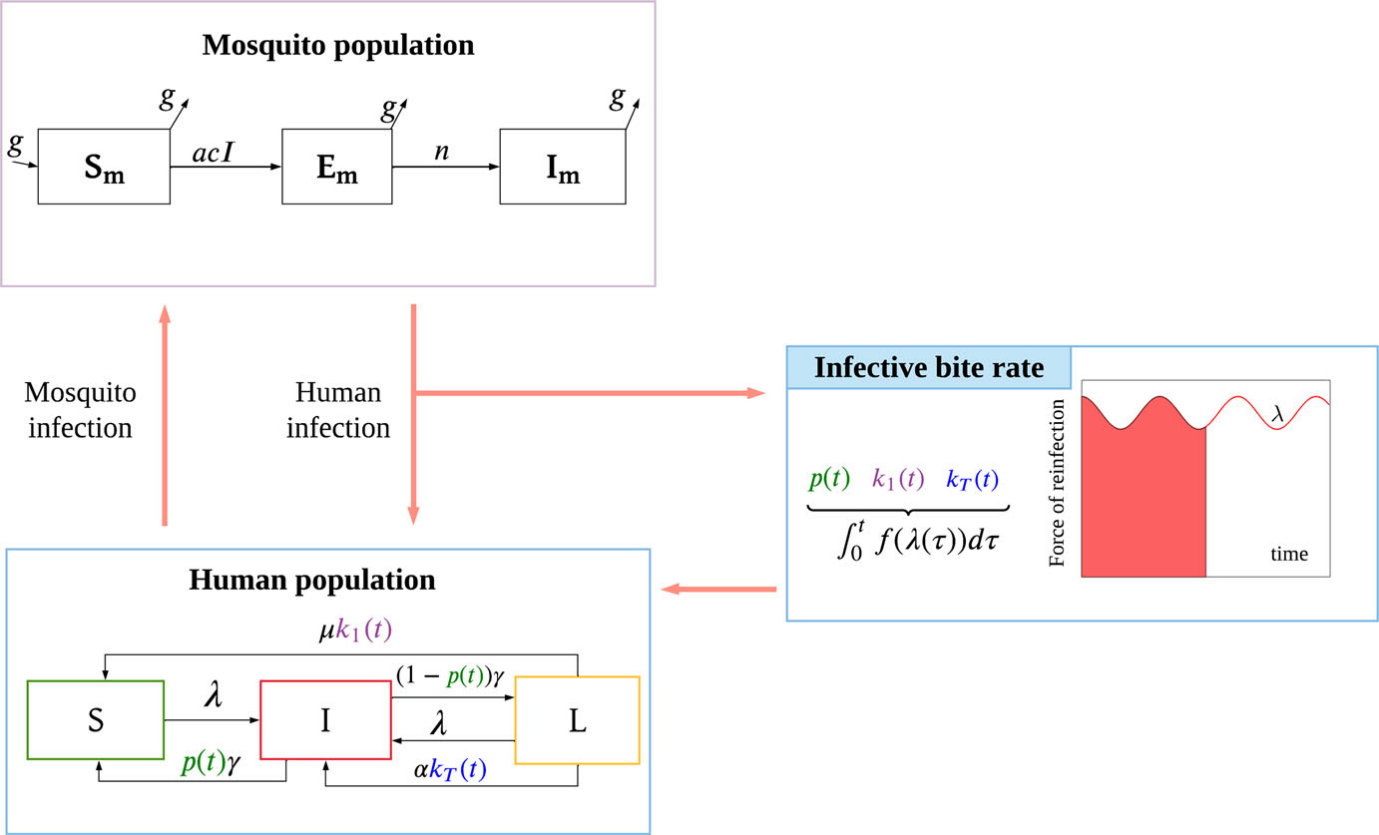
Schematic illustration of the multiscale model. *S*, *I* and *L* represent the fraction of the human population that are susceptible with no hypnozoites, blood-stage infected and liver-stage infected, respectively. Individuals in the *I* compartment may or may not carry hypnozoites. The time-dependent parameters *p*(*t*), $k_1(t)$, and $k_T(t)$ (the probability that blood-stage infected individuals have no hypnozoites, the probability that liver-stage infected individuals have 1 hypnozoite and the expected size of the hypnozoite reservoir in liver-stage infected individuals, respectively) are derived from the within-host model and take into account the history of the force of reinfection, $\lambda (\tau ),$ where $\tau$ is the mosquito bite time and $\tau \in (0, t]$. These together make the multiscale model a system of integro-differential equations. $S_m,\ E_m$, and $I_m$ are the fraction of susceptible, exposed, and infectious mosquitoes, respectively. Other parameters are defined in Table 1

In this section, we develop a multiscale mathematical model for *P. vivax* transmission. In order to enable later extensions to the model, we aim to keep the population-level model as simple as possible. To capture the complex hypnozoite dynamics (as depicted in Fig. 1) at the population level, we embed a within-host model into our population-level model. This is achieved by deriving time-dependent model parameters that are functions of the history of the force of reinfection, which are then fed into the population-level model (for the human species). See Fig. 2 for an overview of the multiscale model.

### Population-Level Model

The human population model consists of three compartments; *S*, *I* and *L* represent the fraction of the human population that are susceptible with no hypnozoites, blood-stage infected and liver-stage infected, respectively. Here we use “liver-stage” infection to refer to individuals with hypnozoites in their liver but without a blood-stage infection. Individuals in the *I* compartment may or may not carry hypnozoites while having a blood-stage infection (see Fig. 1). For the mosquito population, we define $S_m,\ E_m$ and $I_m$ to be the fraction of susceptible, exposed and infectious mosquitoes, respectively; we note that more complicated mosquito dynamics could readily be included in the model. However, for simplicity we adopt only three subpopulations.

Figure 2 is a schematic diagram of the model for the human and mosquito populations and all model parameters are defined in Table 1. Individuals in both *S* and *L* compartments develop new primary blood-stage infections at rate $\lambda (t)=mabI_m(t)$, where $\lambda$ is force of reinfection, defined to be the per-capita infective bite rate from the perspective of each human, *m* is the number of mosquitoes per human, *a* is the mosquito biting rate, and *b* is the transmission probability from mosquito to human. Blood-stage infections are cleared at rate $\gamma$. We define *p*(*t*) as the probability of blood-stage infected individuals having no hypnozoites in their liver. Therefore, individuals that clear their infection return to *S* with probability *p*(*t*) and with probability $1-p(t)$ move to *L*.

From the *L* compartment, individuals move to the susceptible compartment if they have only one hypnozoite remaining (with probability $k_1(t)$) and that hypnozoite dies (each hypnozoite dies independently at a constant rate $\mu$). Hence we define $k_i(t)$ as the probability that a liver-stage infected individual has *i* hypnozoites within their liver. Individuals in the *L* compartment will have a new blood-stage infection when a hypnozoite activates (each hypnozoite activates independently at a constant rate $\alpha$). We define the average number of hypnozoites for liver-stage infected individuals as $k_T(t)=\sum _{i=1}^\infty ik_i(t)$ so that $\alpha k_T(t)$ is the total relapse rate.

**Table 1:** Definitions, values and sources for model parameters. The parameter ranges indicated in square brackets were used in the sensitivity analysis

Symbol	Definition	Value/s	Source
*a*	Biting rate of mosquitoes	0.21 day$^{-1}$ per mosquito	(Garrett-Jones 1964)
*b*	Transmission probability: mosquito to human	0.5	(Smith et al. 2010)
*c*	Transmission probability: human to mosquito	0.23	(Bharti et al. 2006)
*g*	Mosquito death rate	0.1 day$^{-1}$	(Gething et al. 2011)
*m*	Number of mosquitoes per human	0.58 [0, 3]	(White et al. 2014)
*n*	Rate of mosquito sporogony	/12 days$^{-1}$	(Gething et al. 2011)
$\gamma$	Blood-stage infection clearance rate	1/60 day$^{-1}$	(Collins et al. 2003)
$\alpha$	Hypnozoite activation rate	1/332 [0, 1/50]day$^{-1}$	(White et al. 2014)
$\mu$	Hypnozoite death rate	1/425 [0, 1/50] day$^{-1}$	(White et al. 2014)
$\nu$	Average number of hypnozoites per mosquito bite	5 [0, 10]	(White et al. 2016)
$\lambda$	Force of reinfection	Calculated	$\lambda =mabI_m$
*p*	Probability blood-stage infected individual has nohypnozoites within liver	Time varying	Calculated, (Mehra et al. 2022)
$k_i$	Probability liver-stage infected individual has *i*hypnozoites within liver	Time varying	Calculated, (Mehra et al. 2022)
$k_T$	Average number of hypnozoites within liver for liver-stage infected individuals	Time varying	Calculated, (Mehra et al. 2022)

Susceptible mosquitoes become exposed at rate *acI* if they take a blood meal from an infected individual, where *a* is the mosquito bite rate, *c* is the transmission probability from human to mosquitoes, and *I* is the fraction of blood-stage infected individuals. After the incubation period, which has expected duration of 1/*n*, mosquitoes become infectious and can transmit parasites to humans. We assume equal mosquito birth and death rate of *g*.

Using the above assumptions, we write the population-level model equations as:

1 $$\begin{aligned} \frac{\mathrm{d}S}{\mathrm{d}t}=&-\lambda S+\mu k_1(t)L+p(t) \gamma I, \end{aligned}$$

2 $$\begin{aligned} \frac{\mathrm{d}I}{\mathrm{d}t}=&\lambda (S+L)+\alpha k_T(t)L-\gamma I, \end{aligned}$$

3 $$\begin{aligned} \frac{\mathrm{d}L}{\mathrm{d}t}=&-\lambda L-\mu k_1(t)L-\alpha k_T(t)L+(1-p(t))\gamma I, \end{aligned}$$

4 $$\begin{aligned} \frac{\mathrm{d}S_m}{\mathrm{d}t}=&g-acIS_m-gS_m, \end{aligned}$$

5 $$\begin{aligned} \frac{\mathrm{d}E_m}{\mathrm{d}t}=&acIS_m-\left( g+n\right) E_m, \end{aligned}$$

6 $$\begin{aligned} \frac{\mathrm{d}I_m}{\mathrm{d}t}=&nE_m-gI_m, \end{aligned}$$

where $\lambda =mabI_m,\ \text {and}\ k_T(t)=\sum _{i=1}^{\infty } ik_i(t).$ The time-dependent parameters *p*(*t*), $k_1(t)$, and $k_T(t)$ are derived from the within-host model and take into account the history of the force of reinfection, $\lambda (\tau ),\ \text {where}\ \tau \ \text {is the mosquito bite time and}\ \tau \in (0, t]$, making the multiscale model a system of integro-differential equations. Expressions for these parameters will be presented in Sect. 2.2 and are based on the work of Mehra et al. (2022).

### Within-Host Model

**Fig. 3:**
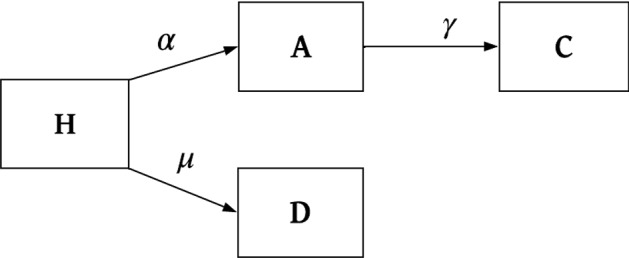
Schematic illustration of within-host model for a single hypnozoite where *H*, *A*, *C*, and *D* represent states of establishment, activation, clearance and death of the hypnozoite, respectively. Parameters $\alpha ,\ \mu$ and $\gamma$ have the same meaning as in population-level model (Table 1). Figure adapted from Mehra et al. (2022) to depict the short-latency phase

Here we consider a special case of the within-host framework of Mehra et al. (2022) which is for short-latency (hypnozoites can immediately activate after establishment without going through a latency phase) strains and in the absence of treatment. The within-host model of Mehra et al. (2022) first considers the dynamics of a single hypnozoite and then allows the establishment of multiple hypnozoites via each infectious bite, with mosquito bites modelled to follow a Poisson process. Each hypnozoite can undergo activation at a constant rate $\alpha$ (which immediately triggers a blood-stage infection that is cleared at a constant rate $\gamma$) or death at a constant rate $\mu$, due to the death of the host liver cell. Let *H*, *A*, *C*, and *D* represent the state of establishment, activation, clearance and death for a single hypnozoite, respectively. Therefore, each hypnozoite that is established (*H*) has two possible final states: death before activation (*D*); or clearance (*C*) that follows activation (*A*) that gives a blood-stage infection (see Fig. 3). Similar within-host dynamics were first introduced by White et al. (2014) without the clearance of relapses resulting from hypnozoite activation. From Equations (13)–(16) in Mehra et al. (2022), the probability mass function (PMF) of the states, ($p_H(t),p_A(t),p_C(t),p_D(t)$), for a hypnozoite established at time $t=0$ is given by

7 $$\begin{aligned} p_H(t)=&e^{-(\alpha +\mu )t}, \end{aligned}$$

8 $$\begin{aligned} p_A(t)=&\frac{\alpha }{(\alpha +\mu )-\gamma }\left( e^{-\gamma t}-e^{-(\alpha +\mu )t}\right) , \end{aligned}$$

9 $$\begin{aligned} p_C(t)=&\frac{\alpha }{\alpha +\mu }\left( 1-e^{-(\alpha +\mu )t}\right) -\frac{\alpha }{(\alpha +\mu )-\gamma }\left( e^{-\gamma t}-e^{-(\alpha +\mu )t}\right) , \end{aligned}$$

10 $$\begin{aligned} p_D(t)=&\frac{\mu }{\alpha +\mu }\left( 1-e^{-(\alpha +\mu )t}\right) , \end{aligned}$$

where $p_f(t)$ represents the probability of the hypnozoite being in state $f \in \{H, A, C, D\}$ at time *t*.

The framework to account for continuous mosquito inoculation was introduced by Mehra et al. (2022), and assumes

- dynamics of hypnozoites are independent and identically distributed, with PMF given by Equations (7)–(10);
- infective mosquito bites follow a non-homogeneous Poisson process with time-dependent rate, or force of reinfection, $\lambda (\tau )$. The mean number of infective bites in the interval (0, *t*], *q*(*t*), is given by
  11 $$\begin{aligned} q(t)=\int _0^t \lambda (\tau )\mathrm{d}\tau ; \end{aligned}$$
- the number of hypnozoites established by each mosquito bite is geometrically distributed (as in White et al. (2014)) with mean $\nu$;
- each infectious bite causes a primary infection which is cleared at rate $\gamma$;
- hypnozoites die only due to the death of the host liver cell at rate $\mu$ (e.g. there is no administration of anti-hypnozoital drugs); and
- individuals are first exposed to infective mosquito bites at time $t=0$.

Let $N_f(t)$ denote the number of hypnozoites in states $f\in \{ H,A,C,D\}\ :=F$ at time *t* and $N_P(t)$, $N_PC(t)$ denote the number of ongoing and cleared primary infections, respectively, at time *t*. Defining the state space $F':=\left\{ H,A,C,D,P,PC\right\}$, the probability generating function (PGF) for

$$\mathbf{N} (t)=(N_H(t),N_A(t),N_C(t),N_D(t),N_P(t),N_{PC})$$

with $\mathbf{N}(0)=\mathbf{0}$ can be written following from Equation (30) in Mehra et al. (2022) (short-latency case ($k=0$) with probability of getting blood-stage infection after an infectious bite, $p_{prim}=1$):

12 $$\begin{aligned}&G(z_H,z_A,z_C,z_D,z_P,z_{PC}):=\mathbb {E}\left[ \displaystyle \prod _{f\in F'} z_f^{N_f(t)}\right] \nonumber \\&\quad =\text{ exp }\left\{ -q(t)+\int _0^t \frac{\lambda (\tau )\left( z_Pe^{-\gamma (t-\tau )}+(1-e^{-\gamma (t-\tau )})z_{PC}\right) }{1+\nu \left( 1-\sum _{f\in F} z_f.p_f(t-\tau )\right) }d\tau \right\} . \end{aligned}$$

We now use the PGF in Eq. (12) to derive expressions for the population-level parameters *p*(*t*), $k_1(t)$, and $k_T(t)$.

#### Probability Blood-Stage Infected Individual has No Hypnozoites: *p*(*t*)

In the population-level model (Eqs. (1)–(6)), *p*(*t*) is defined as the probability that an individual has an empty hypnozoite reservoir conditional on an ongoing blood-stage infection (i.e. primary infection or relapse). That is,

13 $$\begin{aligned} p(t)&=P\big (N_H(t)=0|N_A(t)>0 \cup N_P(t)>0\big )\nonumber \\&=\frac{P(N_H(t)=0)-P(N_H(t)=N_A(t)=N_P(t)=0)}{1-P(N_A(t)=N_P(t)=0)}. \end{aligned}$$

We can use Eq. (12) to determine

14 $$\begin{aligned}&P(N_H(t)=0)\nonumber \\&\quad =\text {Probability that individual has an empty hypnozoite reservoir at time}\ t \nonumber \\&\quad =G(z_H=0, z_A=1, z_C=1, z_D=1, z_P=1, z_{PC}=1)\nonumber \\&\quad =\text {exp}\left\{ -q(t)+\int _0^t \frac{\lambda (\tau )}{1+\nu p_H(t-\tau )}d\tau \right\} , \end{aligned}$$

15 $$\begin{aligned}&P\big (N_A(t)=N_P(t)=0\big )\nonumber \\&\quad =\text {Probability that individual is neither experiencing a relapse nor a}\nonumber \\&\quad \qquad \text {primary infection at time}\ t\ \text {(i.e. no blood-stage infection)}\nonumber \\&\quad =G(z_H=1, z_A=0, z_C=1, z_D=1, z_P=0, z_{PC}=1)\nonumber \\&\quad =\text {exp}\left\{ -q(t)+\int _0^t \frac{\lambda (\tau )(1-e^{-\gamma (t-\tau )})}{1+\nu p_A(t-\tau )}d\tau \right\} , \end{aligned}$$

and

16 $$\begin{aligned}&P\big (N_H(t)=N_A(t)=N_P(t)=0\big )\nonumber \\&\quad =\text {Probability that individual is neither experiencing an infection}\nonumber \\&\quad \qquad \text { nor has any hypnozoites in their liver at time}\ t \nonumber \\&\quad =G(z_H=0, z_A=0, z_C=1, z_D=1, z_P=0, z_{PC}=1)\nonumber \\&\quad =\text {exp}\left\{ -q(t)+\int _0^t \frac{\lambda (\tau )(1-e^{-\gamma (t-\tau )})}{1+\nu (p_H(t-\tau )+p_A(t-\tau ))}d\tau \right\} . \end{aligned}$$

Note that Eqs. (14)–(16) involve integration over the entire history of the force of reinfection, $\lambda (\tau )$ for $\tau \in (0,t]$, and hence in general *p*(*t*) needs to be estimated using numerical integration.

#### Probability Liver-Stage Infected Individual has 1 Hypnozoite in Liver: $k_1(t)$

The probability that a liver-stage infected individual has 1 hypnozoite in the liver at time *t* (that is, the conditional probability for $N_H(t)$ given an individual does not have an ongoing blood-stage infection at time *t*) is

17 $$\begin{aligned} k_1(t)=&P(N_H(t)=1|N_A(t)=N_P(t)=0,N_H(t)>0)\nonumber \\ =&\frac{P(N_H(t)=1|N_A(t)=N_P(t)=0)}{1-P(N_H(t)=0|N_A(t)=N_P(t)=0)}.\nonumber \\ =&\frac{\text {exp}\left\{ g(0,t)-g(1,t)\right\} }{1-P(N_H(t)=0|N_A(t)=N_P(t)=0)}\nonumber \\&\quad \times \int _0^t \frac{\lambda (\tau )(1-e^{-r(t-\tau )})\nu p_H(t-\tau )}{[1+\nu \big (p_H(t-\tau )+p_A(t-\tau )\big )]^2}d\tau , \end{aligned}$$

where the expression for $P(N_H(t)=1|N_A(t)=N_p(t)=0)$ follows from Equation (78) in Mehra et al. (2022) (without treatment) and

$$\begin{aligned} \text {exp}\left\{ g(0,t)-g(1,t)\right\} = \frac{P\big (N_H(t)=N_A(t)=N_P(t)=0\big )}{P\big (N_A(t)=N_P(t)=0\big )}. \end{aligned}$$

Also, $P(N_H(t)=0|N_A(t)=N_p(t)=0)$ is obtained by dividing Eq. (16) by Eq. (15).

#### Average Number Hypnozoites Within Liver-Stage Infected Individuals: $k_T(t)$

The average number of hypnozoites within liver-stage infected individuals, $k_T(t)$, is defined by:

$$\begin{aligned} k_T(t)=\sum _{i=1}^\infty ik_i(t)&= \Big (\frac{\mathbb {E}\left[ N_H(t)|N_A(t)=N_P(t)=0\right] }{1-P(N_H(t)=0|N_A(t)=N_P(t)=0)}\Big ), \end{aligned}$$

where $\mathbb {E}\left[ N_H(t)|N_A(t)=N_P(t)=0\right]$ is the expected size of the hypnozoite reservoir in an uninfected (no blood-stage infection) individual and given by Equation (77) (without treatment and with $p_{prim}=1$) in Mehra et al. (2022). Therefore,

18 $$\begin{aligned} k_T(t)&=\frac{1}{1-P(N_H(t)=0|N_A(t)=N_P(t)=0)}\nonumber \\&\quad \times \int _0^t \left( \frac{\nu \lambda (\tau )\big (1-e^{-r(t-\tau )}\big ) p_H(t-\tau )}{[1+\nu p_A(t-\tau )]^2 }\right) d\tau . \end{aligned}$$

We remark that Eq. (18) allows us to consider a hypnozoite reservoir of infinite size; we do not need to impose a maximum number of hypnozoites, $L_\text {{max}}$ (see Sect. 1). In this way, our model has an advantage over other approaches that need to truncate the size of the reservoir for practical/numerical purposes.

### Numerical Solution of Multiscale Model

To obtain the numerical solution of the model (Eqs. (1)–(6)) over time *t*, we need to evaluate *p*(*t*), $k_1(t)$ and $k_T(t)$ defined in Eqs. (13), (17) and (18), respectively, from the within-host model that depends on the history of the force of reinfection $\lambda (\tau )$, where $\tau \in (0,t]$. Since evaluating *p*(*t*), $k_1(t)$, and $k_T(t)$ involves numerical integration, we implement our own integro-differential equation (IDE) solver, see Algorithm 1. We used a 4th-order Runge–Kutta method to numerically solve the ODEs and the trapezoidal method to evaluate the numerical integration for the parameters in the within-host model (Eqs. (13), (17) and (18)). The within-host model is coupled to the population-level model at each time, *t*, when we evaluate $p(t),\ k_1(t)$, and $k_T(t)$ from the within-host model based on the history of reinfection $\lambda (\tau )$, $\tau \in (0,t]$, to obtain the numerical solution for the population-level model at time $t+\Delta t$ with time increment $\Delta t$. Unless otherwise stated, we have imposed initial conditions of $S(0)=1,\ I(0)=0,\ L(0)=0,\ S_m(0)=0.95,\ E_m(0)=0,$ and $I_m(0)=0.05$. Note that, these initial conditions were chosen for illustrative purposes; the transient dynamics of the model will be different for another choice of initial conditions.
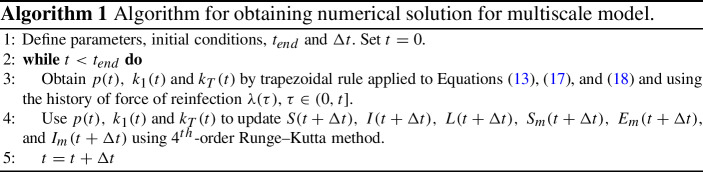

**Fig. 4:**
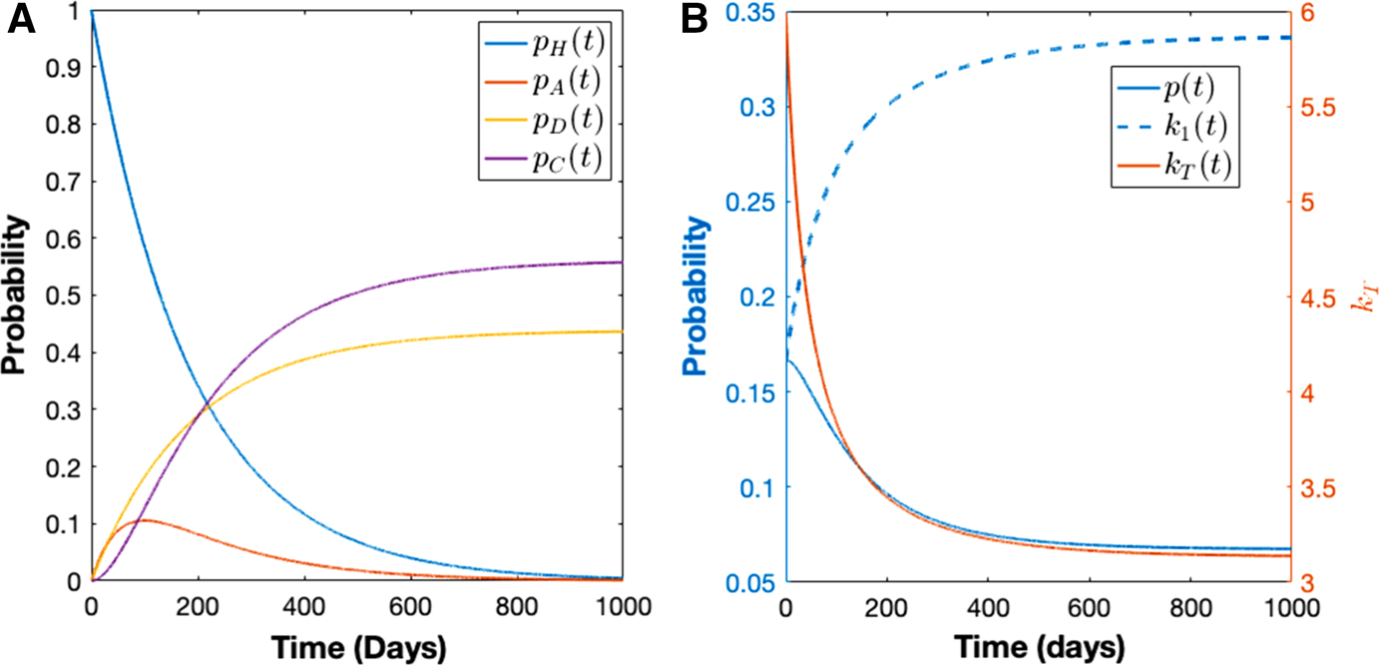
(**A**) PMF for a single hypnozoite with $\big (p_H(0),p_A(0),p_D(0),p_C(0)\big )=(1,0,0,0)$ using Eqs. (7)–(10) where $p_H(t),\ p_A(t)$, $p_D(t), p_C(t)$ represents probability of hypnozoite establishment, activation, death, and clearance at time *t*, respectively. (**B**) Probability of no hypnozoites given blood-stage infection, *p*(*t*) using Eq. (13), probability of 1 hypnozoite given no infection, $k_1(t)$ using Eq. (17), and $k_T(t)$ using Eq. (18). Note the different scale for $k_T$. Here we have used a constant force of reinfection of $\lambda =0.005$ and other parameters are as per Table 1 (Color figure online)

## Results

Using our multiscale model derived in Section 2, we can explore some important features of *P. vivax* transmission dynamics. For example, we can gain insight into disease burden and the hypnozoite reservoir size at time *t*. We can also experiment with different parameters to see their effect on disease transmission. Figure 4 shows results from the within-host model for a constant force of reinfection. Figure 4A depicts the PMF for a single hypnozoite over time (Eqs. (7)–(10)) being in the establishment, activation, death and clearance states over time, *t*. The probability of hypnozoite establishment (blue line) decreases from unity (at $t=0$) over time while the probability of death (yellow line) and clearance (purple line) both increase to nonzero steady states since these are the absorbing states of the system. The probability of hypnozoite activation (red line) rises after establishment and peaks around $t\approx 100$ days. Figure 4B depicts *p*(*t*), $k_1(t)$, and $k_T(t)$ over time. For our choice of parameter values, the probability of having no hypnozoites in the liver given a blood-stage infection, *p*(*t*), decreases over time while the probability of having 1 hypnozoite given a liver-stage infection, $k_1(t)$, increases with time. Meanwhile, the average number of hypnozoites in the liver given a liver-stage infection, $k_T(t)$ decreases over time.

**Fig. 5:**
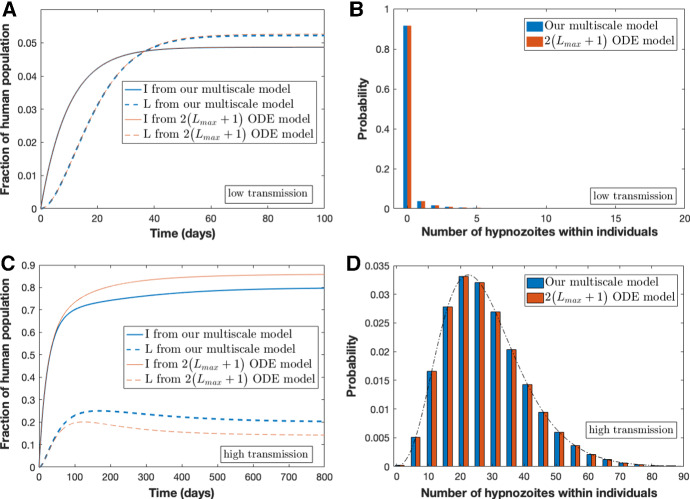
Comparison of results from our multiscale model with those from the $2\big (L_{\mathrm{max}}+1\big )$ ODE model under constant force of reinfection, $\lambda$. Subplots (**A**) and (**C**) illustrate the dynamics based on low transmission and high transmission, respectively. Subplots (**B**) and (**D**) compare the distribution of hypnozoites between our multiscale model (blue) and model containing $2\big (L_{max}+1\big )$ ODEs (red). In (**A**) and (**B**), transmission is low with parameters $\lambda =0.005$, $\gamma =1/10$ day$^{-1}$, $\alpha =1/1000$ day$^{-1}$, $\mu =1/10$ day$^{-1}$ while in (**C**) and (**D**), transmission is high with $\lambda =0.03$ (Color figure online)

In Appendix B.1, we show analytically that our multiscale model, that consists of 3 population-level equations and an embedded within-host submodel, exhibits the same steady state hypnozoite distribution for constant force of reinfection, $\lambda$, as the (countably infinite) ODE model structure in Fig. 1 and adopted in White et al. (2014). We compare the transient dynamics of our multiscale model to the truncated $2\big (L_{max}+1\big )$ ODE model under two scenarios of constant force of reinfection: (i) low transmission with $\lambda =0.005$ in Fig. 5A and B and (ii) high transmission with $\lambda =0.03$ in Fig. 5C and D. Consistent with the analysis presented in Appendix B.1 (see Eqs. (B-20) and (B-25), the steady-state hypnozoite distribution of the two models (regardless of the constant value used for the force of reinfection) is identical (Fig. 5B and D). Figure 5A and C show the fraction blood-stage and liver-stage infected from both models when the force of reinfection is low and high, respectively. Blood-stage and liver-stage infected individuals under the $2\big (L_{\mathrm{max}}+1\big )$ ODE model is obtained by $\sum _{i=0}^{L_{\mathrm{max}}} I_i$ and $\sum _{i=1}^{L_{\mathrm{max}}} S_i$, respectively. For low force of reinfection, the transmission dynamics for the two models agree very closely (Fig. 5A) while for high force of reinfection, the transient dynamics agree closely, but the steady states differ (Fig. 5C). The discrepancy between the models is due to a difference in how super-infection is accounted for at the population level while the underlying within-host model allows for super-infection; for simplicity we have not allowed for individuals to have overlapping infections (see Appendix A). Even when the force of reinfection is time-dependent, $\lambda (t) =mabI_m(t)$, the hypnozoite distribution at steady state from both models is still very similar (see Fig. 9 in Appendix B.3).

**Fig. 6:**
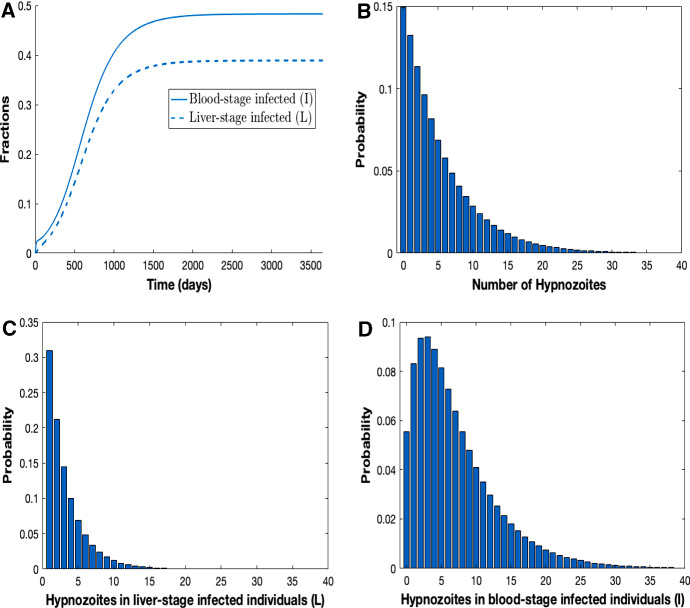
Results from multiscale model for time varying force of reinfection, $\lambda (t)$. Parameters are as per Table 1. Subplot **A** illustrates the fraction of blood-stage (*I*) and liver-stage (*L*) infected individuals over time. Subplot **B** illustrates the hypnozoite distribution in population at steady state (that is, after 3500 days) obtained as per Equations (74)–(75) (without treatment) in Mehra et al. (2022). Subplot **C** illustrates the hypnozoite distribution within liver-stage infected (*L*) individuals at steady state (after 3500 days) obtained as per Equations (78)–(79) (without treatment) in Mehra et al. (2022). Finally, Subplot **D** illustrates the hypnozoite distribution in blood-stage infected (*I*) individuals at steady state (after 3500 days) obtained as per Equation (80) in Mehra et al. (2022)

**Fig. 7:**
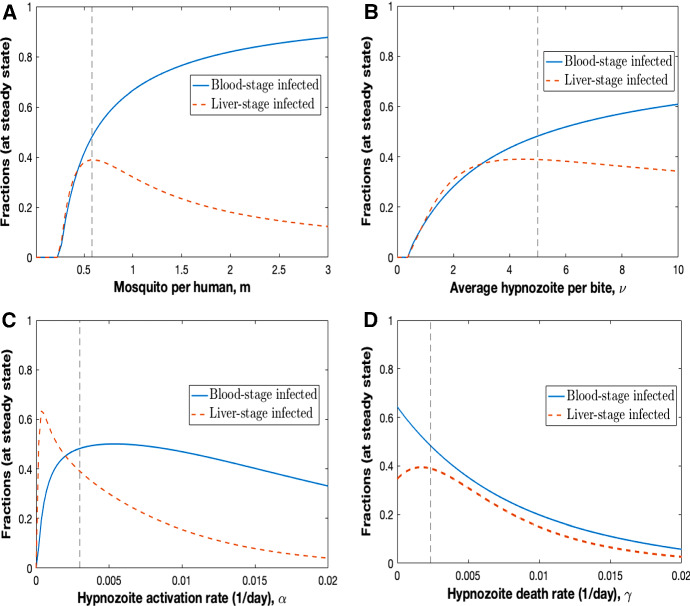
Sensitivity analysis showing the steady state fraction (after running numerical solution for sufficiently long) of blood-stage (*I*) and liver-stage (*L*) infected individuals when model parameters are varied for **A** mosquito per human, *m*; **B** average hypnozoite per bite, $\nu$; **C** hypnozoite activation rate, $\alpha$; and **D** hypnozoite death rate, $\mu$. Vertical lines indicate the parameter value used to generate the results presented in Fig. 6

Figure 6 shows the numerical solution of our multiscale model, for parameter values given in Table 1. The fraction of individuals that are blood-stage infected (*I*) and liver-stage infected (*L*) both increase over time before reaching a steady state (Fig. 6A). Given the choice of parameters, the mode of the hypnozoite distribution is 0 in the long run (Fig. 6B). Figure 6C and D shows the hypnozoite distribution at steady state within liver-stage and blood-stage infected individuals, respectively. At steady state the mode of the hypnozoite distribution for liver-stage infected individuals is 0 while blood-stage infected individuals have a mode of 3 hypnozoites in their liver. The peak number of hypnozoites overall and conditional on infection status (blood-stage or liver-stage) varies with the choice of parameters.

We performed a sensitivity analysis for the model parameters *m*: number of mosquitoes per human, $\nu$: average number of hypnozoites per bite, $\alpha$: hypnozoite activation rate, and $\mu$: hypnozoite death rate (Fig. 7) since these parameters are strong drivers of disease transmission in the multiscale model. Figure 7A shows the fraction of blood-stage (*I*) and liver-stage infected (*L*) individuals at steady state as the number of mosquitoes per human (*m*) varies. When the number of mosquitoes per human is low, there are not enough mosquitoes to sustain disease transmission (e.g. $I=L=0$). As the number of mosquitoes increases past some threshold (depends on choice of other model parameters), a nonzero and increasing proportion of individuals are blood-stage and liver-stage infected at steady state; the blood-stage infected fraction increases monotonically (saturates to unity with increasing *m*) with the number of mosquitoes since the force of reinfection (that is, the probability of infection per unit time) increases with *m* ($\lambda =mabI_m$). However, the fraction of the population that are liver-stage diminishes for extreme values of *m* as individuals are increasingly likely to have a blood-stage infection. Note that blood-stage individuals may or may not have hypnozoites in the liver. Figure 7B shows the fraction of blood-stage and liver-stage infected individuals at steady state as the average number of hypnozoites conferred from a single mosquito bite ($\nu$) varies. When $\nu =0$, no hypnozoites establish in the liver and, as a result, there are no liver-stage infections. For our choice of baseline parameter values (with $\nu =0$) there are insufficient infections to sustain transmission and the blood-stage fraction also approaches zero (disease free equilibrium). The same behaviour is observed for $\nu$ less than some critical threshold. As the average number of hypnozoites per bite passes the critical threshold, the infected fractions (blood-stage and liver-stage) at steady state increase with increasing number of hypnozoites per bite as disease transmission is sustained. As $\nu$ increases further, the blood-stage fraction continues to increase as relapses are increasingly common. However, the liver-stage fraction at steady state decreases with increasing $\nu$ as individuals are more likely to have a blood-stage infection.

Figure 7C depicts the fraction of blood-stage and liver-stage infected individuals at steady state as the hypnozoite activation rate ($\alpha$) varies. When $\alpha =0$, the liver-stage infected fraction is zero since there are no relapses, only primary infections. The blood-stage infected fraction is also zero for our choice of baseline parameters with $\alpha =0$ (the system goes to the disease free equilibrium). As the activation rate increases, the fraction of liver-stage infected individuals rises sharply but this trend cannot be sustained as increasing the hypnozoite activation rate further leads to increased blood-stage infections (converted from liver-stage infections due to hypnozoite activation). For large values of the hypnozoite activation rate, both blood-stage and liver-stage infected fractions will decrease with higher activation rate $\alpha$ since higher activation rates yield overlapping relapses. The batch of hypnozoites established by a bite will activate shortly after inoculation, giving rise to relapses that overlap with the primary infection with super-infection not being taken into account in our population-level model. As $\alpha \rightarrow \infty$, for our choice of baseline parameters, both blood-stage and liver-stage fractions tend to zero; hypnozoites activate immediately after establishment and hence coincide with the primary infection which is the same as if only considering primary infections. Figure 7D shows the fraction of blood-stage and liver-stage infected individuals at steady state as the hypnozoite death rate ($\mu$) varies. When $\mu =0$, hypnozoites can only activate and hence the fraction of blood-stage infected individuals is maximal. As $\mu$ increases, the fraction of blood-stage infected individuals decreases; there are fewer blood-stage relapses since hypnozoites are more likely to die prior to activation. For our choice of baseline parameters (see Table 1), the fraction of liver-stage infected individuals increases when $\mu$ is increased from small values. As the hypnozoite death rate is increased further, the liver-stage infected fraction decreases with increasing $\mu$ because the hypnozoite reservoir diminishes; hypnozoites die quickly and individuals will become susceptible.

We can use the results presented in Fig. 7 to provide epidemiological insights into the benefit of *P. vivax* control measures that effect the four parameters varied in the sensitivity analysis. For example, the use of insecticide-treated nets (ITNs), indoor residual spraying (IRS), and long-lasting insecticide-treated nets (LLINs) can reduce the number of mosquitoes (Bowen 2013; Hawley et al. 2003; Gari and Lindtjørn 2018). Our sensitivity analysis predicts that, under our multiscale model, if we can reduce the force of reinfection ($\lambda (t) =mabI_m(t)$) enough, such as by reducing the number of mosquitoes per human (*m*; Fig. 7A), we can eliminate *P. vivax* disease transmission.

## Discussion

*P. vivax* transmission is known to be largely driven by the hypnozoite reservoir (Wells et al. 2010). Hence, it is important to account for the complexity of the size and variation in the hypnozoite distribution in population-level mathematical models of the disease. Despite this, most existing transmission models for *P. vivax* (Águas et al. 2008; Ishikawa et al. 2003; Roy et al. 2013; Silal et al. 2019) over-simplify the hypnozoite reservoir into a single additional compartment. Exceptions to this is the work by White et al. (2018, 2014) which either uses a large number of compartments to capture the complexity of the size and variation in the hypnozoite distribution or adopts an agent-based approach. While these are both valid modelling approaches, the resulting models are already considerably detailed (e.g. $2\big (L_{max}+1\big )$ ODEs), meaning that extension to include other important factors may be difficult.

In this paper, we have embedded a within-host model (Mehra et al. 2022) in a population-level model for *P. vivax*. By keeping the population-level model simple, while capturing the complicated hypnozoite within-host dynamics, extension of the model to include other important factors will be feasible. For example, *P. vivax* dynamics are complicated by disease transmission as it can be dependent on age in some cases; studies in PNG have shown higher prevalence of *P. vivax* in children aged between 2 and 5 years (Genton et al. 2008). Furthermore, anti-hypnozoital drugs (e.g. primaquine and tafenoquine) are not recommended for pregnant and/or lactating people, and those with G6PD deficiency (Howes et al. 2012; Watson et al. 2018), meaning that each of them are further potential factors to consider.

We use differential equations at the population level that govern the proportion of the population with different infection status (susceptible, blood-stage infection, liver-stage infection). These differential equations depend on three time-dependent parameters, namely, the probability that a blood-stage infected individual has no hypnozoites within their liver, *p*(*t*); the probability that a liver-stage infected individual has 1 hypnozoite within their liver, $k_1(t)$; and the average number of hypnozoites for liver-stage infected individuals, $k_T(t)$. Each of these parameters was derived as a function of the history of the force of reinfection, based on work of Mehra et al. (2022), in the form of a definite integral. The resulting multiscale model was therefore a system of IDEs that was solved numerically (Sect. 2.3).

Our multiscale model was used to gain insights into important features such as disease dynamics and hypnozoite distribution(s) within blood-stage and liver-stage infected individuals (Fig. 6). Furthermore, our sensitivity analysis revealed that if we can reduce the force of reinfection by using ITNs, IRS and/or LLINs to reduce the number of mosquitoes per human, we can eliminate *P. vivax* disease transmission (Fig. 7).

**Fig. 9:**
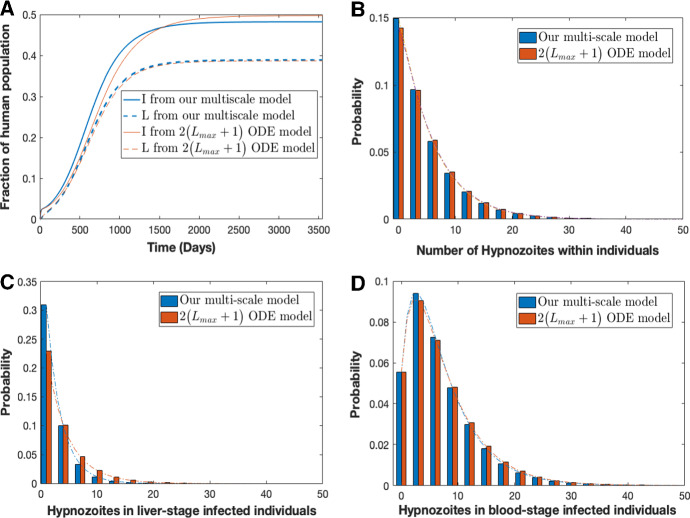
Comparison of results from our multiscale model and the $2\big (L_{max}+1\big )$ ODE model under time varying force of reinfection, $\lambda (t)$. Subplot **A** shows the fractions of blood-stage and liver-stage infected individuals over time. Blood-stage and liver-stage infected individuals for the $2\big (L_{max}+1\big )$ ODE model is obtained by $\sum _{i=0}^{L_{max}} I_i$ and $\sum _{i=1}^{L_{max}} S_i$, respectively. Subplot **B** depicts the hypnozoite distribution in population at steady state (that is, after 3500 days) obtained as per Equations (74)–(75) (without treatment) in Mehra et al. (2022). Subplot **C** shows the Hypnozoite distribution within liver-stage infected individuals at steady state (after 3500 days) obtained as per Equations (78)–(79) (without treatment) in Mehra et al. (2022). Subplot **D** shows the Hypnozoite distribution in blood-stage infected (*I*) individuals at steady state (after 3500 days) obtained as per Equation (80) in Mehra et al. (2022). In Subplot **B**, **C**, and **D**, the bars are for consecutive intervals of 3 hypnozoites and the dashed lines show the whole distribution. All parameters are as per Table 1

Importantly, we have been able to show analytically that our multiscale model exhibits an identical steady-state hypnozoite distribution for constant force of reinfection, $\lambda$, as the ODE model structure shown in Fig. 1 and presented in White et al. (2014); infection dynamics at the population level are indistinguishable for a low transmission setting but appreciably different for a high transmission context (Fig. 5) due to differences in how super-infection is accounted for at the population level. Interestingly, even under time-dependent force of reinfection, the hypnozoite distribution at steady state from both models is still similar and the prevalence of infection is comparable under certain parameter regimes (Fig. 9). Our model has the advantage that the population-level component is considerably simpler than the $2\big (L_{\mathrm{max}}+1\big )$ ODEs of White et al. (2014) and we also avoid the need to artificially truncate at $L_{\mathrm{max}}$ hypnozoites.

The framework that we have introduced here can be extended in several ways. Firstly, it is well-positioned for extension to include factors such as age, pregnancy and G6PD deficiency status. Furthermore, our multiscale model does not consider important features such as immunity, heterogeneity in bite exposure, and seasonality; which are all avenues for future work. It is still not clear exactly what causes hypnozoites to activate (Mueller et al. 2009) although there are hypotheses around recognition of a mosquito protein (Hulden and Hulden 2011) and febrile illness (Imwong et al. 2007; White et al. 2014), both of which our model does not consider. We also ignored disease-induced death in our model as a significant amount of malaria-related death is due to *P. falciparum* (WHO 2020) but this will be included in future iterations of the model.

In order to progress towards elimination of *P. vivax*, it will be vitally important to target the hypnozoite reservoir, as around 80% of infections are attributed to relapses from activating hypnozoites (Robinson et al. 2015). Since our multiscale model can capture the effect of the hypnozoite reservoir in disease transmission, it provides a platform to study *P. vivax* disease elimination. The model provides a baseline epidemiological framework to examine disease transmission and elimination strategies including mosquito control (e.g. ITNs, IRS and LLINs). With appropriate extensions, for example to incorporate G6PD structure, our model can help in evaluating *P. vivax* anti-hypnozoital drugs administered, for example, under radical cure regimens.

## Figures and Tables

**Fig. 8:**
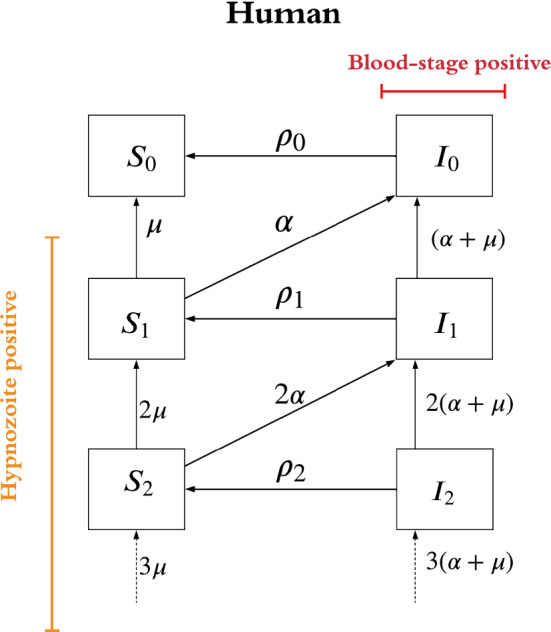
Schematic diagram of White et al. (2014) model, reproduced with permission. Here $S_i$ represents the fraction of the human population that are susceptible with *i* hypnozoites and $I_i$ represents the fraction of the human population that have a blood-stage infection with *i* hypnozoites. Parameters are as in Table 1

## Data Availability

Data sharing is not applicable to this article as no datasets were generated or analysed during the current study. MATLAB code to reproduce all the Figures can be accessed from GitHub https://github.com/n-anwar/multiscale_model_Figures.git and provides an overview of how we have implemented Algorithm 1.

## Super-Infection

Super-infection in malaria occurs when an individual has more than one blood-stage infection at a given time (Smith et al. 2010). In reference to *P. vivax*, individuals can have hypnozoites in the liver even after recovery of their blood-stage infection. So, another pathway to super-infection for *P. vivax* is when individuals with a blood-stage infection undergo another infection due to relapsing hypnozoites (Portugal et al. 2011; Smith et al. 2010). The modelling assumption of super-infection, which was made by Macdonald (Macdonald 1950), considers that individuals can start further infections while having an existing infection and that one infection does not change the infectious period of another. For example, in White et al. (2014) (Fig. 8), super-infection is modelled through a recovery rate given by

A-19 $$\begin{aligned} \rho _i=\dfrac{\lambda +i\alpha }{\exp (\frac{\lambda +i\alpha }{\gamma })-1}, \end{aligned}$$

where *i* is the number of hypnozoites, $\lambda$ is the force of reinfection, $\alpha$ is the hypnozoite activation rate, and $\gamma$ is the recovery rate. Without super-infection, the recovery rate is just $\rho _i=\gamma$.

## Comparison with White et al. (2014) Model

To the best of our knowledge, the only population-level *P. vivax* transmission models that have considered hypnozoite variation are developed by White et al. through a compartment modelling approach (White et al. 2014) (see Figure 8) or an agent-based approach (White et al. 2018). The compartment-based approach (White et al. 2014) allows super-infection that delays the time to recovery from infection (Eq. (A-19)) and considers an infinite number of hypnozoites (truncated at a maximum of $L_{max}$ hypnozoites for numerical purposes). Note that as described in Sect. 2.2.3, our model does not require this artificial truncation.

### PGF for Steady-State Hypnozoite Distribution Under Constant $\lambda$

#### Our Multiscale Model

From Equation (37) in Mehra et al. (2022), it follows that the PGF for the size of the hypnozoite reservoir at steady state ($N_H^*$) under constant force of reinfection, $\lambda$, for our multiscale model is the PGF of the negative binomial distribution

$$\begin{aligned} N^*_B\sim \text {NegativeBinomial} \left( \frac{\nu }{1+\nu },\frac{\lambda }{\alpha +\mu }\right) \end{aligned}$$

with PMF

B-20 $$\begin{aligned} P(N_H^*=i)=\frac{\Gamma \left( \frac{\lambda }{\alpha +\mu }+i\right) }{i!\cdot \Gamma \left( \frac{\lambda }{\alpha +\mu }\right) }\frac{\nu ^i}{(1+\nu )^{i+\frac{\lambda }{\alpha +\mu }}}, \end{aligned}$$

where $\Gamma$ is the Gamma function.

#### White et al. (2014) Model

The system of ODEs for the White et al. (2014) model is given by

B-21 $$\begin{aligned} \frac{dS_i}{dt}=&-\lambda S_i-i(\alpha +\mu )S_i+(i+1)\mu S_{i+1}+\rho _iI_i\qquad \qquad i \in \mathbb {Z}_{\ge 0}, \end{aligned}$$

B-22 $$\begin{aligned} \frac{dI_i}{dt}=&-\lambda I_i+\sum _{j=0}^{i} \lambda _{j\rightarrow i}\left( S_j+I_j\right) -i(\alpha +\mu )I_i+(i+1)(\alpha +\mu )I_{i+1}\nonumber \\ {}&+(i+1)\alpha S_{i+1}-\rho _iI_i\qquad i \in \mathbb {Z}_{\ge 0}, \end{aligned}$$

where $S_i$ and $I_i$ represents the fraction of susceptible and infected individuals with *i* number of hypnozoites, respectively, and $\rho _i$ is given in Eq. (A-19) and

$$\begin{aligned} \lambda _{j\rightarrow i}=&\lambda \left( \frac{\nu }{\nu +1}\right) ^{i-j}\frac{1}{\nu +1}. \end{aligned}$$

Assuming steady-state transmission, that is $\lambda$ is constant over time, let $G_S$ and $G_I$ be the PGFs for state *S* and *I*, respectively; namely:

$$\begin{aligned} G_S(Z,t)=&\sum _{i=0}^\infty S_iZ^i,\ \ Z\in [0,1],\\ G_I(Z,t)=&\sum _{i=0}^\infty I_iZ^i,\ \ Z\in [0,1]. \end{aligned}$$

Now multiplying Eq. (B-21) by $Z^i$ and taking the summation from 0 to $\infty$, we get

$$\begin{aligned} \frac{\partial G_S}{\partial t}&=\sum _{i=0}^\infty \frac{d}{dt}(S_iZ^i)=-\lambda G_S-(\alpha +\mu )Z\frac{\partial G_S}{\partial Z}+\mu \frac{\partial G_S}{\partial Z}+\sum _{i=0}^\infty \rho _iI_iZ^i. \end{aligned}$$

Similarly,

$$\begin{aligned} \frac{\partial G_I}{\partial t}=\sum _{i=0}^\infty \frac{d}{dt}(I_iZ^i)&=-\lambda G_I+\frac{\lambda }{1+\nu (1-Z)}\Big (G_S+G_I\Big )-(\alpha +\mu )Z\frac{\partial G_I }{\partial Z}\nonumber \\ {}&+(\alpha +\mu )\frac{\partial G_I }{\partial Z}+\alpha \frac{\partial G_S }{\partial Z} -\sum _{i=0}^\infty \rho _iI_iZ^i. \end{aligned}$$

At steady state $(\frac{\partial G_S}{\partial t}=\frac{\partial G_I}{\partial t}=0$) we have

B-23 $$\begin{aligned}&\left( (\alpha +\mu )Z-\mu \right) \frac{d G_S }{d Z}=-\lambda G_S+\sum _{i=0}^\infty \rho _iI_iZ^i , \end{aligned}$$

B-24 $$\begin{aligned}&(\alpha +\mu )(Z-1)\frac{d G_I}{d Z}-\alpha \frac{d G_S }{d Z}=-\lambda G_I-\sum _{i=0}^\infty \rho _iI_iZ^i +\frac{\lambda }{1+\nu (1-Z)}\Big (G_S+G_I\Big ). \end{aligned}$$

Let $M_i=S_i+I_i$ and $G_M=G_S+G_I$. Now adding Eqs. (B-23) and (B-24) gives

$$\begin{aligned}&(\alpha +\mu )Z\frac{dG_M}{dZ}-(\alpha +\mu )\frac{dG_M}{dZ}=-\lambda G_M+\frac{\lambda }{1+\nu (1-Z)}G_M. \end{aligned}$$

Now solve the ODE with $G_M(1,0)=1$ to give

$$\begin{aligned} G_M=\left( 1+\nu (1-Z)\right) ^{-\lambda /(\alpha +\mu )}. \end{aligned}$$

Hence, the hypnozoite distribution at steady state from the White et al. model is

B-25 $$\begin{aligned} P(M=i)&=\frac{G_M^i(0)}{i!} =\left( \frac{\nu }{1+\nu }\right) ^{i+\frac{\lambda }{\alpha +\mu }}\left( \frac{\nu ^{\frac{-\lambda }{\alpha +\mu }}}{i!}\right) \frac{\Gamma \left( \frac{\lambda }{\alpha +\mu }+i\right) }{\Gamma \left( \frac{\lambda }{\alpha +\mu }\right) }. \end{aligned}$$

From Eqs. (B-20) and (B-25) we can see that the distribution of hypnozoites at steady state from both models is the same under constant $\lambda$ .

### Comparison Under Time-Dependent Force of Reinfection, $\lambda$

Figure 9 shows a comparison of results from our multiscale model and the $2\big (L_{\mathrm{max}}+1\big )$ ODE model of White et al. (2014). Figure 9A shows the fraction of blood-stage and liver-stage infected individuals from both models. Blood-stage and liver-stage infected individuals from the $2\big (L_{\mathrm{max}}+1\big )$ ODE model is obtained by $\sum _{i=0}^{L_{\mathrm{max}}} I_i$ and $\sum _{i=1}^{L_{\mathrm{max}}} S_i$, respectively. Both models suggest that individuals are most likely to have 0 (mode is 0) hypnozoites within their liver (Fig. 9B). In addition, both models suggest that blood-stage infected individuals are most likely to have 3 (mode is 3) hypnozoites within their liver (Fig. 9D) although our multiscale model suggests a slightly higher probability.
